# Amplified injustices and mutual aid in the COVID-19 pandemic

**DOI:** 10.1177/1473325020973326

**Published:** 2021-03

**Authors:** Finn McLafferty Bell

**Affiliations:** School of Social Work, University of Michigan, Ann Arbor, USA

**Keywords:** Carers, community work, disasters, environmental social work, resilience, uncertainty

## Abstract

The COVID-19 pandemic has amplified existing injustices in the United States, which is exemplified in Ypsilanti, Michigan. However, the pandemic also provides an opportunity to re-imagine existing ways of being in the world, and mutual aid networks that have provided for people's basic needs during multiple crises while also working towards more radical change provide an opportunity for social workers to examine their relationship to “helping.” The author uses their personal experience with a local mutual aid network to examine the power and possibility of mutual aid, particularly in times of crisis, as well as sources of social work resistance to decentralized and non-professional forms of helping and caring. These lessons are carried beyond the COVID-19 pandemic to their consequences for the looming climate crisis.

“Every crisis, actual or impending, needs to be viewed as an opportunity to bring about profound changes in our society. Going beyond protest organizing, visionary organizing begins by creating images and stories of the future that help us imagine and create alternatives to the existing system.”

– Grace Lee Boggs (2012: xxi)

As a community social worker, my first action when the COVID-19 pandemic hit my area in March 2020 was to reach out to the most vulnerable people around me and to connect with the people who were best positioned to help. I left notes on the doors of my neighbors who I knew to be elders, frail, or live alone, as well as on the door of an anarchist collective. I knew the collective had been active in creating a mutual aid network—the organized practice of “people giv[ing] what they can and get[ting] what they need” (Shepard in Izlar, 2019: 352)—as well as conducting community trainings around the United States on Mutual Aid Disaster Relief, which I had attended. My previous research on disasters led me to suspect that the most effective responses would come from ordinary people on the ground who were coming together to help, and I wanted to contribute what I was able. In addition to the global pandemic, we also simultaneously had food shortages, a 500-year flood, and racist police violence within our state. While all of this has been overwhelming, the mutual aid approach of directly meeting people’s needs by sharing resources while organizing for more radical change has been effective in meeting social work goals in the middle of a chaotic time and deserves examination. As Arundhati Roy (2020) so eloquently stated, “the pandemic is a portal” and we should take this crisis as an opportunity for transformation in how we do social work.

I knew that my home of Ypsilanti, Michigan, USA—a working-class deindustrialized city of 21,000 that is 35 miles west of Detroit and adjacent to the much wealthier and whiter Ann Arbor—would face many challenges in a disaster, as so many residents already live precariously. Disasters amplify existing injustices. This was brought home to me one day in late March when I went into Ann Arbor and passed a grocery store, where I saw a very neat line of dozens of shoppers, with each person masked and standing six feet apart. As I drove back to Ypsilanti, I saw a similar line but rather than waiting for groceries, people were waiting to “donate” their blood plasma for cash. At the time of this writing (July 2020), the COVID-19 case data on our county shows clear disparities: 49.7% of the total cases in Washtenaw county occurred in Ypsilanti’s two zip codes (Washtenaw County Health Department, 2020), despite those zip codes including only 28.8% of the county’s population in the 2010 census (Zip-codes.com, 2020). Further, as is true all over the US, there is racial disparity in who is impacted. In a county in which African Americans only make up 12.3% of the population, 33% of COVID cases, 41% of hospitalizations, and 30% of deaths have been African Americans (Washtenaw County Health Department, 2020). While white people in Washtenaw County experienced COVID-19 at a rate of 25 per 10,000, African Americans had a rate of 88 per 10,000 (Oppel et al., 2020). Soberingly, 1 in 1500 African Americans in the US have died as a result of this pandemic (Pilkington and Milman, 2020).

The COVID-19 pandemic has demystified the racial capitalism undergirding the US and put the deadly consequences of an economy created in the fires of slavery and settler colonialism on full display (Robinson, 1983; Sharpe, 2016). It took a pandemic for us to finally deem the low-paid workers who provide our food, care for the sick and elders, and dispose of our waste to be deemed “essential.” And, these essential workers are disproportionately women and people of color in the US (Powell, 2020). The additional exposure to the virus that essential workers and their families face compounded with environmental racism and inequitable access to food and healthcare makes the virus far more deadly for people of color in the US. Washtenaw County is one of the most unequal counties in the US, and COVID-19 has amplified this injustice, triggering a long-overdue reckoning on racism as a public health crisis (Rigg, 2020).

By reaching out to the neighbors involved in the mutual aid network, my partner and I were able to plug into myriad ways to help the people most impacted in our community. Prior to the pandemic, our neighbors had asked us about cutting down the fence that separates our properties. As avid gardeners who spend most free time in our backyard, we were hesitant about the loss of privacy. However, in the new normal of the pandemic, not having that fence has transformed our relationship, not just with our neighbors, but with community more broadly. Rather than being lone gardeners, we have started to see our work as part of a collective of care. We pass eggs from our chickens, produce that we grow or buy from local farmers, and seedlings across the driveway for them to distribute to food insecure neighbors through the food bank they run at a local church.

Like many people throughout the US, we started sewing face masks for healthcare workers during shortages of personal protective equipment (PPE) due to the perversions of just-in-time capitalism (Moody, 2020). When it became clear that community members also should be wearing masks, the mutual aid network started distributing them at the food bank. In late May, after video footage of a local sheriff’s deputy punching an African American woman in the head surfaced (Dodge, 2020), the mutual aid network provided support to organizers of ongoing protests in defense of Black Lives, including distributing masks, water, and basic hygiene supplies. Their support was key to safely mobilizing people in an area that was still hit hard by the pandemic. When extremely heavy rains fell across Michigan in May, with the Midland area receiving 4.7 inches in 48 hours which caused the failure of two major dams (Cappucci and Freedman, 2020), the mutual aid network asked us to sew masks made from HEPA vacuum bags that would protect people responding to the flood from mold exposure. The ability of mutual aid networks to quickly shift gears to respond to the needs of community members is one of their strengths, particularly when compared to bureaucratic social service organizations.

Mutual aid is seldom discussed as a social work method (Izlar, 2019), but it is an important part of how oppressed communities have long ensured their “collective survival,” by using strategies of caring for and protecting each other to build resilience to both spectacular and everyday disasters (Bell et al., 2019). As we face more and more crises in more and more places under a changing climate, mutual aid networks that focus on “direct action and direct services” (Shepard, 2015: 79) provide a model for how to effectively meet social work goals of providing social services to people in need while simultaneously working to address the root causes of injustice.

Izlar (2019) suggests that part of social work’s resistance to mutual aid is our professional identity as helpers and the deprofessionalizing implications of embracing mutual aid, which anyone can do, as a social work practice. While my partner and I freely offered help, we were stumped when the neighbors continually asked what needs they could help us to meet. Part of us being stumped was our class and racial privilege—together, we still had a steady income and a social safety net, as well as white socialization that trained us to seek self-sufficiency. Part of it was that we legitimately did not want to take resources away from people who needed them more than us. However, another large part of our resistance to being “helped” was our professional identities, as a social worker (me) and a librarian (my partner).

The conflict between my resistance to receiving help despite being committed to giving it caused me to experience perplexity—“a state of being … which encourages the recognition of uncertainty, honors the dissonance between past assumptions and new understandings, and creates opportunities for meaningful relationships, personal growth, and social reform” (Chappell Deckert and Koenig, 2019: 164). I was suddenly questioning my own motivations and history: Was my helping a form of charity if it was unidirectional? What could I learn from my younger anarchist neighbors in how to be in true solidarity with our community? Further, what could social workers learn if we were to uphold our values without being so attached to our professional identity as helpers and the hierarchical relationship it necessitates?

These questions are particularly relevant as we enter a new era of uncertainty and chaos. The COVID-19 pandemic has thrown many of us into a state of perplexity as so many aspects of our lives have been disrupted and we are uncertain if and when they will return to normal. There has been so much loss—foremost the loss of life that has been disproportionately felt in communities of color, but also the loss of a sense of safety, of health, of connection, of community, of place, and of routine. For all of the extreme disruption and grief that COVID-19 has caused, this particular pandemic likely will end sooner rather than later. On the other hand, we are entering an era of climate chaos, with no end in sight. Climate change will continue to provide extreme disruptions and disasters in communities all over the world for the foreseeable future, with the most vulnerable communities being hit first and worst. It will likely overwhelm our social welfare infrastructure, as well as our supply chains, institutions, and ways of life (Brulle and Norgaard, 2019). At this particular time in history, understanding a decentralized method of meeting people’s basic survival needs while simultaneously challenging injustice and building alternative ways of being in the world that are more liberatory could not be more relevant and timely.

Izlar (2019) argues that it is not until social workers personally find themselves in need that they will come to embrace mutual aid as a social work method, using social workers under Greek austerity as an example. I have escaped the worst impacts of the pandemic thus far; however, when I did have a COVID-19 scare, knowing that there was a mutual aid network in my backyard was certainly reassuring. A challenge for me, and perhaps for social work more broadly, is how can we embrace and build infrastructure for more horizontal, anti-authoritarian models of caring before we are in direct need of them? Doing so not only makes us more resilient to the impacts of all kinds of disasters, but it also makes our lives and our communities better in the here and now. In this way, it represents a prefigurative politics that helps us to create the world that we want to live in. Knowing that climate change is likely to further amplify injustices, how can social workers promote prefigurative politics that facilitate the collective survival strategies that communities have long used to endure exploitation and oppression (Bell et al., 2019)?

In *A Paradise Built in Hell*, Rebecca Solnit (2009) writes about the “extraordinary communities that arise in disasters” when suddenly the world breaks open and anything is possible. People rise to the occasion, and in these most frightening and grief-stricken of days, they find more meaning than in all of their other days combined. I have glimpsed these extraordinary communities during this pandemic in my own city through the mutual aid network as well as the other ways that people are caring for and protecting each other.

Solnit (2009) further outlines the usually anti-climactic end of these extraordinary communities: the professionals come in and thank all of the volunteers and promptly dismiss them. All of that liberatory possibility, all of that meaning-making in the worst of circumstances, that new way of being in the world goes up in bureaucratic smoke. Is that what will happen this time? Precedent suggests so. However, a global pandemic is not just any disaster. Part of what makes it so deadly is that systems everywhere are being overwhelmed. Usually, other areas send resources to the site of the disaster, including those bureaucrats who take over. But, in this case, the disaster is everywhere (Moody, 2020). While this has been deadly in terms of the distribution of necessary medical equipment and personnel, it also means that the groups currently caring for their communities are likely going to be needed in the long haul, particularly as further disasters arise under a changing climate. Social workers can and should be a part of these groups, but if my experience is illustrative, in order to do so, we need to let go of many of our assumptions about who we are, and tear down the fence that keeps us separated from the rest of the community that is also doing the work, sometimes more effectively than we are.

## References

[bibr1-1473325020973326] Bell FM Dennis MK Krings A (2019) Collective survival strategies and anti-colonial practice in ecosocial work. Journal of Community Practice 27(3–4): 279–295.

[bibr2-1473325020973326] Brulle RJ Norgaard KM (2019) Avoiding cultural trauma: Climate change and social inertia. Environmental Politics 28(5): 886–908.

[bibr3-1473325020973326] Boggs GL (2012) The Next American Revolution. Berkeley: University of California Press.

[bibr4-1473325020973326] Cappucci M Freedman A (2020) Michigan dams fail near midland; catastrophic flooding underway. Washington Post. Available at: www.washingtonpost.com/weather/2020/05/20/michigan-dams-fail-midland/ (accessed 6 July 2020).

[bibr5-1473325020973326] Chappell Deckert J Koenig TL (2019) Social work perplexity: Dissonance, uncertainty, and growth in Kazakhstan. Qualitative Social Work 18(2): 163–178.

[bibr6-1473325020973326] Dodge S (2020) Video of Washtenaw county deputy punching woman sparks outrage in Ypsilanti township. MLive. Available at: www.mlive.com/news/ann-arbor/2020/05/video-of-washtenaw-county-deputy-punching-woman-sparks-outrage-in-ypsilanti-township.html (accessed 6 July 2020).

[bibr7-1473325020973326] Izlar J (2019) Radical social welfare and anti-authoritarian mutual aid. Critical and Radical Social Work 7(3): 349–366.

[bibr8-1473325020973326] Moody K (2020) How “just-in-time” capitalism spread COVID-19: Trade routes, transmission, and international solidarity. Spectre. Available at: https://spectrejournal.com/how-just-in-time-capitalism-spread-covid-19/ (accessed 6 July 2020).

[bibr9-1473325020973326] Oppel RA Gebeloff R Lai KKR, et al. (2020) The fullest look yet at the racial inequity of Coronavirus. *The New York Times*. Available at: https://nyti.ms/3f286n9 (accessed 6 July 2020).

[bibr10-1473325020973326] Pilkington E Milman O (2020) ‘Trump is in a dreamland’: What the US should do now on COVID-19. *The Guardian*. Available at: www.theguardian.com/world/2020/jul/05/coronavirus-us-surge-experts-what-to-do (accessed 6 July 2020).

[bibr11-1473325020973326] Powell C (2020) The color and gender of COVID-19: Essential workers, not disposable people *Think Global Health*. Available at: www.thinkglobalhealth.org/article/color-and-gender-covid-19-essential-workers-not-disposable-people (accessed 6 July 2020).

[bibr12-1473325020973326] Rigg S (2020) For many Ypsi-area residents, recognition of racism as a public health issue is long overdue *Concentrate Media*. Available at: www.secondwavemedia.com/concentrate/features/racismpublichealth0554.aspx (accessed 6 July 2020).

[bibr13-1473325020973326] Robinson CJ (1983) Black Marxism. London: Zed Press.

[bibr14-1473325020973326] Roy A (2020) The pandemic is a portal *Financial Times*. Available at: www.ft.com/content/10d8f5e8-74eb-11ea-95fe-fcd274e920ca (accessed 6 July 2020).

[bibr15-1473325020973326] Sharpe C (2016) In the Wake. Durham: Duke University Press.

[bibr16-1473325020973326] Shepard B (2015) Community Projects as Social Activism. London: Sage.

[bibr17-1473325020973326] Solnit R (2009) A Paradise Built in Hell. New York: Penguin Books.

[bibr18-1473325020973326] Washtenaw County Health Department (2020) COVID-19 cases in Washtenaw County. *Washtenaw County*. Available at: www.washtenaw.org/3108/Cases (accessed 6 July 2020).

[bibr19-1473325020973326] *Zip-codes.com* (2020) Washtenaw County, MI Zip Codes Available at: www.zip-codes.com/county/mi-washtenaw.asp (accessed 6 July 2020).

